# Hot Deformation and Dynamic Recrystallisation Behaviour of Twin-Roll Cast Mg-6.8Y-2.5Zn-0.4Zr Magnesium Alloy

**DOI:** 10.3390/ma14020307

**Published:** 2021-01-08

**Authors:** Madlen Ullmann, Kristina Kittner, Ulrich Prahl

**Affiliations:** Institute of Metal Forming, Technische Universität Bergakademie Freiberg, Bernhard-von-Cotta-Straße 4, 09599 Freiberg, Germany; kristina.kittner@imf.tu-freiberg.de (K.K.); ulrich.prahl@imf.tu-freiberg.de (U.P.)

**Keywords:** flow curve, dynamic recrystallisation, processing map, LPSO phase, Mg-6.8Y-2.5Zn-0.4Zr, twin-roll casting

## Abstract

In this work, the deformation behaviour of a twin-roll cast (TRC) Mg-6.8Y-2.5Zn-0.4Zr alloy during plane strain compression was characterised by high-temperature testing. Based on the experimental data, the values of strain-rate sensitivity, the efficiency of power dissipation and the instability parameter were investigated under the conditions of various hot deformation parameters. In contrast to conventionally cast material, no lamellae of the LPSO (long period stacking ordered) phase were precipitated in the magnesium matrix after TRC. The precipitation of fine lamellar LPSO phases only occurred during cooling to forming temperature after the heat treatment. Dynamic recrystallization (DRX) hardly occurred during deformation at temperatures between 350 °C and 400 °C. This can be attributed to the precipitation of the lamellar LSPO phases, which contribute to retardation of the DRX process. At higher deformation temperatures and strain rates DRX is pronounced and the twin-induced (TRDX) as well as continuous dynamic recrystallization could be identified as the dominant softening mechanisms. The processing maps were established by superimposing the instability map over the power dissipation map, this being associated with microstructural evolution analysis in the hot deformation processes. Two instability zones could be recognised for the twin-roll cast and heat-treated Mg-6.8Y-2.5Zn-0.4Zr alloy: (1) 350 °C to 460 °C and 0.01 s^−1^ to 0.3 s^−1^ and (2) 485 °C to 525 °C and 2.5 s^−1^ to 10 s^−1^, where deformation is not favourable.

## 1. Introduction

Wrought magnesium alloys of the Mg-Y-Zn alloying system are becoming increasingly important because of their high strength at both room temperature and elevated temperatures. The high strength of such alloys is attributed to the presence of long-period stacking ordered phases (LPSO), which are characterised by the fact that the basal planes are enriched with Y and Zn at periodic intervals [1,2]. These ordered structures can have different stacking sequences: 10H [3,4], 12R [5,6], 14H [1,7,8], 18R [1,7,8,9] and 24R [10]. Investigations have shown that LPSO phases have a significant influence on hot deformation behaviour and dynamic recrystallisation. Chen et al. (2020) [11] investigate the importance of the LPSO phase in a Mg-8.02Y-1.99Zn alloy during dynamic recrystallisation under various strain conditions. They find that the alloy exhibits a flow behaviour comparable to that of conventionally available magnesium alloys, but that the LPSO phase significantly influences dynamic recrystallisation (DRX). (I) Kink deformation in LPSO phases are initiation points for DRX. (II) Fragmentation of the LPSO phase favours DRX through particle-stimulated nucleation (PSN). (III) DRX grains are also pinned by the LPSO phases at higher temperatures, and grain boundary migration is inhibited.

LPSO-containing alloys and, in particular, Mg-Y-Zn and Mg-Gd-Zn systems, have been investigated in the past to determine their hot-working behaviour [11,12,13,14,15,16]. The aim is to define parameters for hot-working processes and to understand the mechanisms involved in hot working and DRX. By means of compression tests in the temperature range from 300 °C to 500 °C and strain rates between 0.001 s^−1^ and 10 s^−1^, the hardening and softening behaviour of various LPSO-containing alloys was analysed and described. Based on these results, processing maps were derived that provided information on areas of stable and unstable deformation [17]. For a Mg-9Gd-2.9Y-1.9Zn-0.4Zr alloy as the initial cast material, Xu et al. (2016) [15] determine that 450 °C and 0.001 s^−1^ are optimal hot-working parameters. For a Mg-5.8Y-2.0Zn-0.3Zr alloy (wt.%), Lv et al. (2013) [18] suggest values from 400 °C to 500 °C and 0.001 s^−1^ to 0.01 s^−1^ as well as 500 °C and 0.1 s^−1^ to 1 s^−1^ as optimal hot-working conditions. It is common to all investigations carried out so far that conventionally cast or extruded starting material was examined. However, twin-roll cast strips of LPSO-containing magnesium alloys have not been considered to date. Twin-roll casting enables the production of magnesium strips in an economic and energy-efficient way. Over the past 25 years, intensive research has been conducted on TRC of magnesium alloys and the process has been transferred to industrial production. The most common magnesium alloy AZ31 is well investigated. However, the area of application is limited due to their poor performance at elevated temperatures. The twin-roll casting of the Mg-6.8Y-2.5Zn-0.4Zr alloy offers the possibility to provide strips of a high strength and thermally stable magnesium alloy. Due to the special solidification conditions and the resulting microstructural changes involved, the hot-working behaviour of other initial conditions cannot be assigned to the TRC state. Accordingly, the aim of this work was to investigate the hot working behaviour of a twin-roll cast Mg-6.8Y-2.5Zn-0.4Zr alloy. As a function of the forming temperature and strain rate, flow curves were recorded and, based on these data, the dynamic recrystallisation behaviour was described. Furthermore, process maps were derived which were then used to provide optimal parameters for hot working of the twin-roll cast and heat-treated Mg-6.8Y-2.5Zn-0.4Zr alloy.

## 2. Materials and Methods

The chemical composition of the Mg-6.8Y-2.5Zn-0.4Zr alloy is shown in Table 1. Details on the production of twin-roll cast strips of Mg-6.8Y-2.5Zn-0.4Zr alloy are described in detail in [19].

With a length of 20 mm and a width of 30 mm, samples for compression tests were cut from the twin-roll cast sheets (sheet thickness 5.3 mm). Subsequently, plane strain compression tests were performed on a servo-hydraulic hot-working simulator. A graphite-oil mixture was used as a lubricant to reduce the friction between the punch and sample. Before compression, the samples were heat treated at 500 °C for 2 h. While taking into account friction and dissipation heat, isothermal flow curves were determined from the recorded force-displacement data. In addition, the flow curves were corrected according to Siebel with a coefficient of friction of *µ* = 0.12. Furthermore, softening processes due to the temperature increase as a result of the dissipated forming energy were considered numerically by the thermodynamic temperature factor *K_ϑ_* = exp(*−m*_1_ *× ϑ*), with *m*_1_ = −0.00427 °C^−1^. The forming temperature (350–525 °C) was set by means of an air circulation furnace and maintained for 15 min to ensure the uniform temperature distribution in the sample. Afterwards, continuous compression was performed at strain rates of 0.01 s^−1^, 0.1 s^−1^, 1 s^−1^ and 10 s^−1^. The samples were deformed to an equivalent logarithmic strain of *φ_v_* = 1. At least three tests were performed for each forming condition to ensure a sufficient level of statistical certainty. Immediately after compression, the samples were quenched in water and metallographically prepared for microstructural analysis. All metallographic samples were made as longitudinal sections across the cross-section of the twin-roll cast strip. To this end, the samples were ground and then polished with an oxide polishing suspension. Etching was carried out in two steps. After brief rinsing with a 3% initial solution, the sample was etched with a picric acid solution consisting of 70 mL ethanol, 10 mL distilled water, 10 mL glacial acetic acid and 4.2 g picric acid.

Microstructural analysis was carried out on the compressed and quenched samples at specific locations where the local comparative logarithmic strain corresponded to the total equivalent logarithmic strain. Light microscopic images were taken on a Keyence VHX 6000 microscope at the Institute of Metal Forming, Freiberg, Germany. Based on these images, the recrystallised portion was determined by means of point analysis. Scanning electron microscopic evaluation was then performed on a ZEISS GeminiSEM 450 device at the Institute of Metal Forming, Freiberg, Germany.

Processing maps are based on dynamic material models (DMM) and are used to optimise the hot-deformation parameters. The principles for creating processing maps can be found in [20] and [21], among others. The method takes into account the opposing relationship between the rate of heat generation induced by the forming process and the rate of energy dissipation. The microstructural changes in the structure such as recovery and dynamic recrystallisation as well as material damage are considered. Kocks-Mecking plot was used to describe the dynamic recrystallisation during hot deformation. For each flow curve the strain hardening *θ* *= δk_f_/δφ* or *θ* *=* Δ*k_f_/*Δ*φ* is plotted of the flow stress *k_f_.* Further details of the determination of the critical stress *k_fc_* and the associated critical strain *φ_c_* are given in [22]. The dynamically recrystallised fraction is determined for each set of flow curves (the determined flow curve and the calculated curve for dynamic recovery) [22] according to Equation (1), where *k_f_* *_DRX_* *_ss_* is the stationary flow stress and *φ* *_ss_* the corresponding equivalent strain.

(1) $$X_{dyn}=\frac{k_{f\;DRV}\left(\varphi \right)-k_{f\;DRX}\left(\varphi \right)}{k_{fDRV\;ss}-k_{\;f\;DRX\;ss}}.$$

## 3. Results and Discussion

### 3.1. Microstructure of Twin-Roll Cast and Heat-Treated Alloy

**Figure 1:**
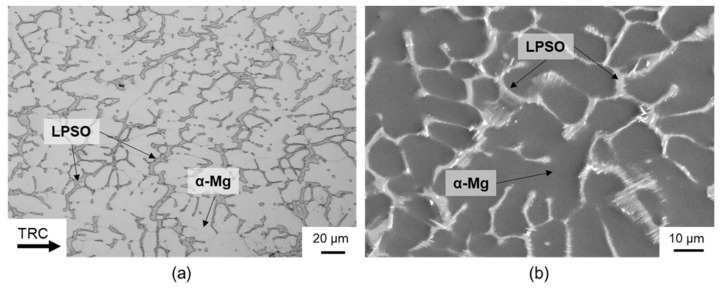
(**a**) Optical and (**b**) SEM micrographs (SE, 15 kV) of the twin-roll cast and heat-treated (500 °C, 2 h) Mg-6.8Y-2.5Zn-0.4Zr alloy.

Figure 1 shows the microstructure of the twin-roll cast Mg-6.8Y-2.5Zn-0.4Zr alloy after heat treatment at 500 °C for 2 h. The microstructure after twin-roll casting consisted primarily of α-magnesium matrix material, which was pervaded by a fine network of LPSO phases. A detailed description of the microstructural composition and the texture of twin-roll cast strips of Mg-6.8Y-2.5Zn-0.4Zr alloy can be found in [19]. On the basis of X-ray diffraction investigations, the LPSO phases that occurred after twin-roll casting could be assigned to the 18R or 14H structures. In comparison to the initial cast material, one significant difference in the microstructural composition of the TRC strips was that in addition to the α-magnesium matrix and the network-like LPSO phase within the matrix, fine lamellar LPSO phases did not occur. After heat treatment at 500 °C with a holding time of 2 h, no changes could be observed in the material’s microstructural composition. The network-like LPSO phases remained intact. Higher temperatures (525 °C) and/or longer holding times (6 h) are required to dissolve such phases [19].

### 3.2. Flow Behaviour

**Figure 2:**
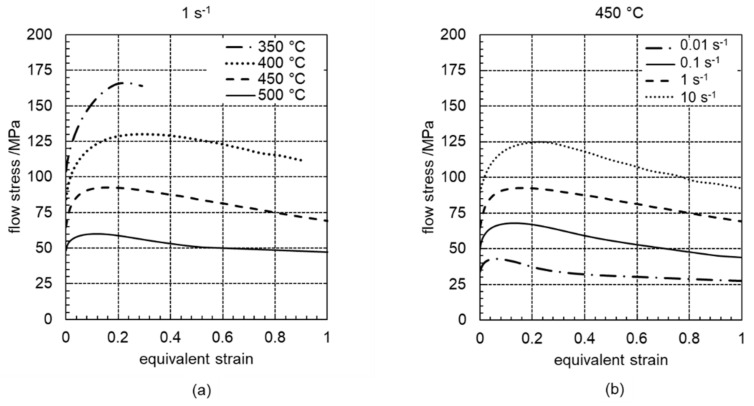
Flow curves of the twin-roll cast and heat-treated Mg-6.8Y-2.5Zn-0.4Zr alloy: (**a**) temperature range between 350 °C and 500 °C for a strain rate of 1 s^−1^ and (**b**) for different strain rates of 0.01 s^−1^, 0.1 s^−1^, 1 s^−1^ and 10 s^−1^ at 450 °C.

Figure 2 summarises the flow curves of the twin-roll cast and heat-treated Mg-6.8Y-2.5Zn-0.4Zr alloy for a temperature range from 350 °C to 500 °C and strain rates of 0.01 s^−1^, 0.1 s^−1^, 1 s^−1^ and 10 s^−1^. It is clear that the hardening and softening behaviour was dependent on both the forming temperature and the strain rate. The results shown here are typical flow curves for the occurrence of dynamic recrystallisation during hot working [23]. With increasing the strain rate and decreasing temperature, an increase in yield strength could be observed. This phenomenon was associated with the fact that the rate of dislocation multiplication increases with increasing the strain rate, the dislocation movement is stopped and, consequently, the yield strength is increased. After the increase of the flow stress in the course of hardening, which was caused by the increase in dislocation density, the flow curves exhibited different progressions depending on the temperature up to the yield stress maximum. From temperatures of 450 °C, the flow stress decreased continuously after reaching the maximum. The decrease in flow stress could be attributed here to the occurrence of dynamic recrystallisation. High temperatures (500 °C) or low strain rates (0.01 s^−1^) achieved the steady state range in the equivalent strain range under consideration (equivalent strain to 1). This meant that complete dynamic recrystallisation had taken place.

Softening processes could hardly be observed at low forming temperatures (350 °C). Here, the flow stress increased continuously because of the increasing hardening. Only a slight decrease of the flow stress can be found after reaching peak stress, which points to the occurrence of softening processes. Flow instabilities caused premature material failure through the occurrence of cracks. Comparable hardening and softening behaviour is determined for a cast Mg-6.8Y-2.5Zn-0.4Zr alloy in [21]. However, comparison with the material investigated in the current study clearly shows that dynamic recrystallisation in the case of the twin-roll cast and heat-treated Mg-6.8Y-2.5Zn-0.4Zr alloy already commenced at lower temperatures. In addition to an earlier onset of DRX (low critical degree of deformation), the results in Figure 2 show that DRX was more likely to have been completed than would be the case for a conventionally cast initial material [21]. It was assumed that DRX was favoured in the twin-roll cast and heat-treated Mg-6.8Y-2.5Zn-0.4Zr alloy. In contrast to the conventionally cast initial material, there were no lamellae precipitated in the magnesium matrix in the twin-roll cast and heat-treated initial state. As already shown in [24], such lamellae can contribute to the delay of the DRX process.

### 3.3. Analysis of Hot Deformation Behaviour of Twin-Roll Cast and Heat-Treated Mg-6.8Y-2.5Zn-0.4Zr Alloy

For the evaluation of the efficiency index *η* and the instability parameter $\xi \left(\dot{\varepsilon }\right)$ as a function of the forming temperature, the logarithmic strain and the comparative strain rate in the process charts, the characteristics of the plastic flow were calculated based on the hot flow curves determined. The mechanisms during hot working were derived based on Equation (2) according to Sellars and Tegart [25,26].

(2) $$\;\dot{\varepsilon }\;=\;A\;{\left[\sin h\left(\alpha \sigma \right)\right]}^{n\;}\exp\left(-\frac{Q}{R\cdot T}\right)$$

**Figure 3:**
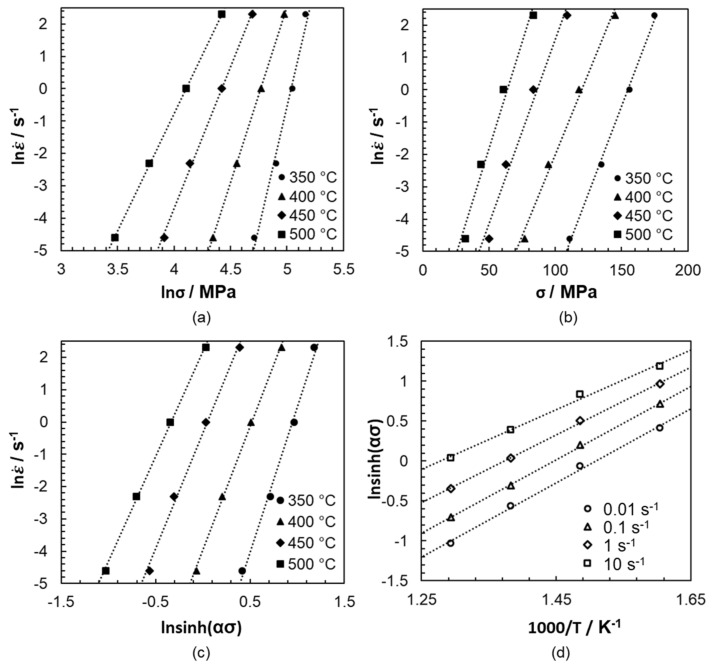
Relationship between $\ln\left(\dot{\varepsilon }\right)$ and (**a**) ln*σ* and (**b**) *σ* as a function of the forming temperature as well as (**c**) $\ln(\dot{\varepsilon )}$ and ln[sinh(*ασ*)] at different forming temperatures and (**d**) ln(sinh(*ασ*)]^−1^/*T* as a function of the strain rate.

The equation takes into account the strain rate $\dot{\varepsilon }$, the material constant *A* at 2.617 × 10^19^ s^−1^, *n* as the creep and stress exponent at 7.487, α as the coefficient of adaptation at 0.0108 MPa^−1^, σ as the maximum stress, *Q* as the average activation energy for plastic flow at 270.63 kJ/mol, the gas constant *R* at 8.314 J/(mol K) and *T* as the thermodynamic forming temperature. The parameters of the equation were determined using the mean values from the increases in the graphs, which represented the linear relationship of the flow stress to the comparative strain rate and temperature (Figure 3). The calculated model coefficients were valid for forming temperatures of between 350 °C and 500 °C and for comparative strain rates of between 0.01 s^−1^ and 10 s^−1^. The calculation was based on the assumption that the processes taking place during forming were diffusion controlled. Accordingly, all model coefficients were temperature- and strain rate-dependent.

The activation energy is a measure of the difficulty of initiating deformations during hot working, and is simultaneously influenced by the dynamic precipitation formation, the pinning effects of dislocations and the occurrence of secondary phases. For cast magnesium alloys with LPSO phases, the activation energy varies between 182 kJ/mol and 276 kJ/mol [27]. The value determined for the twin-roll cast and heat-treated Mg-6.8Y-2.5Zn-0.4Zr alloy was in the upper range at 270 kJ/mol, which was significantly higher than that for grain boundaries (92 kJ/mol) or lattice diffusions (135 kJ/mol) in magnesium [28,29]. The higher value for the activation energy was likely associated with the increased proportion of LPSO phases. These impede dislocation movement during deformation and cause self-diffusion within the crystal lattice to be suppressed [2,30,31].

The model coefficients determined were also used to calculate the Zener–Hollomon parameter *Z* (Equation (3)).

(3) $$Z=\dot{\varepsilon }\;\exp\;\left(\frac{Q}{RT}\right)$$

As a function of *Z* and $\ln\left[\sin h\left(\alpha \sigma \right)\right]$ the power-law relationship is investigated. The linear regression was determined at low Z values with an accuracy of (*R*^2^) = 0.99, resulting in a good approximation of the hyperbolic sine function. According to the constitutive equation (Equation (4))

(4) $${Z\;=\;A\;\sin h}^{n}\left(\alpha \;\sigma \right)$$

**Figure 4:**
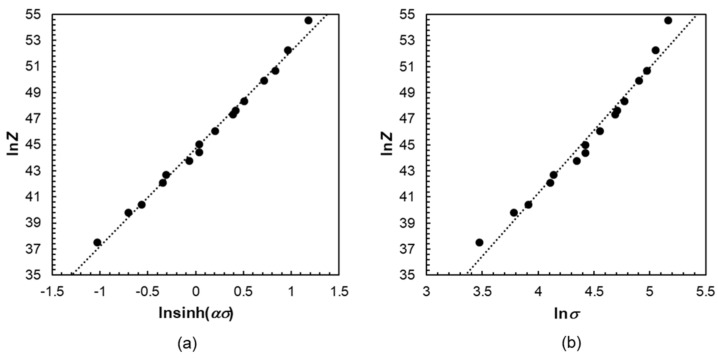
Relationship between the Zener–Hollomon–Parameter and the maximum stress during deformation at high temperatures: (**a**) lnZ–ln[sinh(*ασ*)] and (**b**) lnZ–ln*σ*.

The stress exponent *n* amount to 7.487. The fitting parameter *α* = 0.0108 MPa^−1^ can be determined from the diagram in Figure 4a. In the characteristic hyperbolic sine function, as expected, there is an improved degree of linearity compared to the curves seen in Figure 4b.

### 3.4. DRX Behaviour of Twin-Roll Cast and Heat-Treated Mg-6.8Y-2.5Zn-0.4Zr Alloy

**Figure 5:**
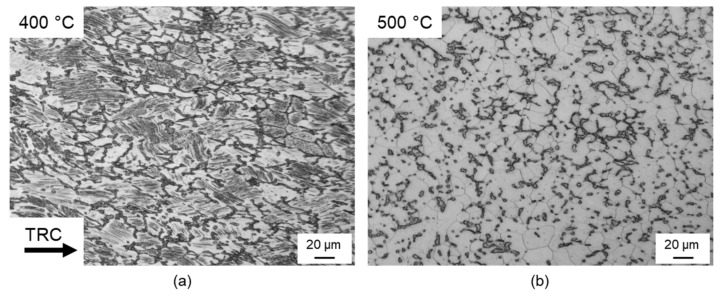
Light microscopic images after plane strain compression at a strain rate of 1 s^−1^ and an equivalent strain of 1: (**a**) 400 °C and (**b**) 500 °C.

**Figure 6:**
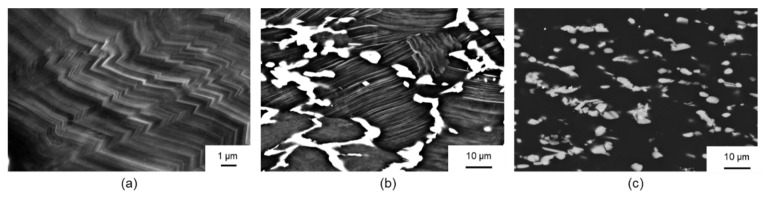
SEM microscopic images after plane strain compression at a strain rate of 1 s^−1^ and an equivalent strain of 1: (**a**) kink band formation of the lamellar LPSO phase at 350 °C, (**b**) at 350 °C showing lamellar LPSO phase and (**c**) at 500 °C without lamellar LPSO phase (BSD, 20 kV).

Figure 5 shows light microscopic images of the Mg-6.8Y-2.5Zn-0.4Zr alloy after plane strain compression at 400 °C and 500 °C at a strain rate of 1 s^−1^ and an equivalent strain of 1. Because of the cooling from a heat-treatment temperature of 500 °C to a forming temperature of 400 °C, fine lamellae precipitated within the α-magnesium matrix (Figure 5a). Dynamic recrystallisation hardly occurred during forming at 400 °C, so that even at an equivalent strain of 1, only a small proportion of recrystallised grains could be observed. One possible reason for this was the precipitation of lamellar LPSO phases (Figure 6b), which contribute to retardation of the DRX process [24]. In contrast, an almost completely recrystallised microstructure was present after forming at 500 °C (Figure 5b). Lamellar precipitations do not occur under these forming conditions (Figure 6c), so that suppression of the dynamic recrystallisation processes was not to be expected.

**Figure 7:**
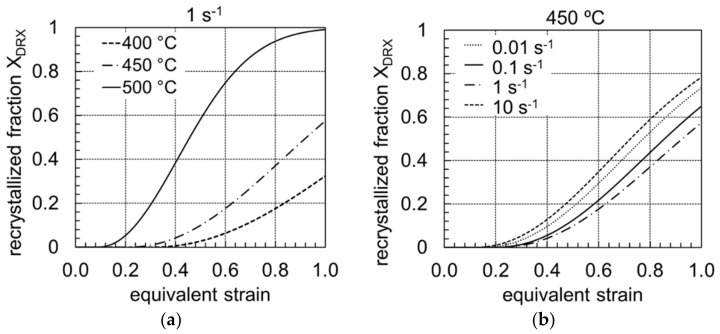
DRX volume fraction of twin-roll cast and heat-treated Mg-6.8Y-2.5Zn-0.4Zr alloy at (**a**) a strain rate of 1 s^−1^ and different temperatures and (**b**) a temperature of 450 °C and different strain rates.

The observations from the microstructural analysis were confirmed by comparison of the recrystallised portions as a function of the forming temperature (Figure 7a) and the strain rate (Figure 7b). The kinetics of DRX can predict the evolution of the microstructure and volume fraction of DRX in terms of S-curves, which is usually expressed by an Avrami equation. The kinetics of DRX evolutions for the investigated alloy is expressed as (Equation (5)):

(5) $$X_{dyn}=1-\exp\left[-1.68\cdot {\left(\frac{\varphi -\varphi_{c}}{\varphi_{0.5}-\varphi_{c}}\right)}^{2.1}\right]$$

where *X_dyn_* is the dynamic recrystallisation integral, *φ* is strain, *φ_c_* is the critical strain and *φ*_0.5_ is the strain at a recrystallised fraction of 50%.

On the basis of the kinetics of DRX, the effects of deformation temperature, strain and strain rate on the DRX volume fraction are shown in Figure 6. It can be seen that the deformation strain required for the same amount of DRX volume fraction increases with decreasing the deformation temperature for a strain rate of 0.01 to 10 s^−1^, which means that DRX was delayed to a longer time period at a lower deformation temperature. In contrast, there was no clear dependence on the strain rate, which was attributed to the change of the DRX mechanism in association with the LPSO phases. The mechanism of DRX and microstructural evolution at different deformation temperatures and strain rates is discussed in part.

Comparison of the recrystallised portion between the forming temperatures 400 °C and 500 °C (at a strain rate of 1 s^−1^) clearly showed that much higher equivalent strains were required to achieve the same recrystallised portions at 400 °C. At an equivalent strain of 1, the sample formed at 500 °C exhibited an almost completely recrystallised microstructure. At 400 °C, the recrystallised portion lay at approx. 30%. It was therefore assumed that higher temperatures favoured dynamic recrystallisation. As an example, Figure 6b shows the influence of the strain rate on the recrystallised portion for a forming temperature of 450 °C. It becomes clear that the higher the strain rate in the range of 0.01 s^−1^ to 1 s^−1^, the higher the equivalent strain required to achieve a certain recrystallised portion. At a strain rate of 0.01 s^−1^, for example, a 50% recrystallised microstructure is achieved at an equivalent strain of 0.75, and at 1 s^−1^ only at 0.9. For example, comparable results are reported for conventionally cast initial material by Kwak et al. (2015) [32] for a Mg-5Y-2.5Zn-1.2Ca alloy and by Lv et al. (2014) [33] for a Mg-5.8Y-2.0Zn-0.3Zr alloy. Higher strain rates (10 s^−1^) cause the DRX process to accelerate. A degree of deformation of 0.7 is sufficient for the production of a 50% recrystallised microstructure.

**Figure 8:**
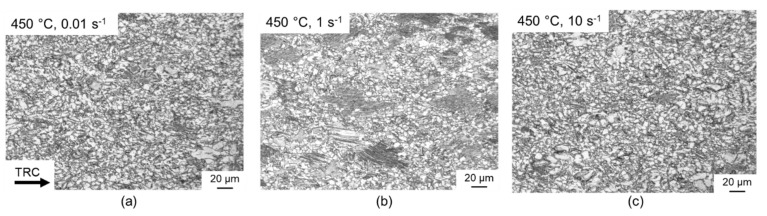
Optical micrographs of samples deformed at 450 °C at different strain rates: (**a**) 0.01 s^−1^, (**b**) 1 s^−1^ und (**c**) 10 s^−1^.

Comparison of the light microscopic images in Figure 8 shows the extent of dynamic recrystallisation as a function of the strain rate in the range of 0.01 s^−1^ to 10 s^−1^ at a forming temperature of 450 °C and an equivalent strain of 1. The dependence of the recrystallised portion on the strain rate can also be observed here. Many small recrystallised grains are visible at a strain rate of 0.01 s^−1^, the proportion of which amounts to as much as 80%. With increasing strain rates of up to 1 s^−1^, this percentage drops to 65% and increases again to a recrystallised portion of 80% at high strain rates of 10 s^−1^.

### 3.5. The Mechanism of DRX

**Figure 9:**
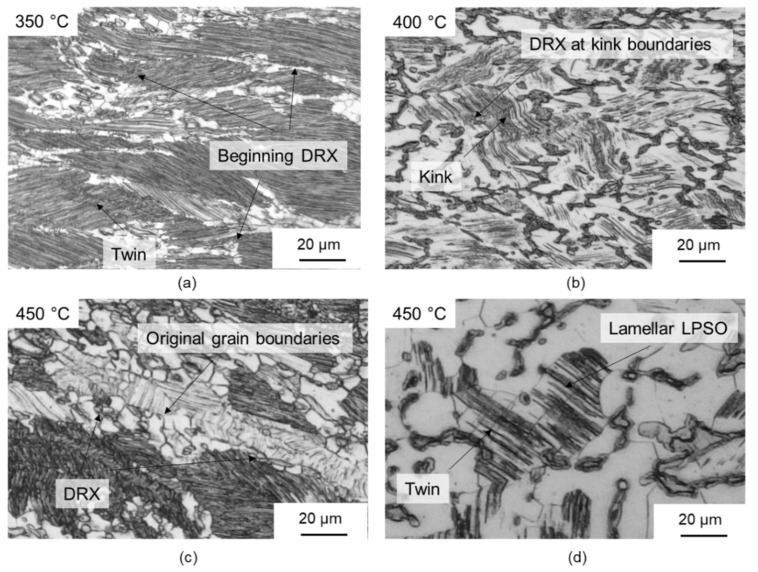
Optical micrographs of samples deformed at different temperatures and strain rates: (**a**) 350 °C, (**b**) 400 °C, (**c**) 450 °C, DRX at original grain boundaries and (**d**) 450 °C, twins together with lamellar LPSO phase.

Because of the high thermal stability of the network-like LPSO phases and the precipitation of fine lamellar LPSO phases (Figure 6b) during cooling to the forming temperature, the forming process and the mechanisms of dynamic recrystallisation are strongly influenced by the different LPSO phases. Figure 9a shows the microstructure after forming at 350 °C. Because of the high differential between the heat-treatment and forming temperatures, fine lamellar LPSO phases precipitate in the grains of the magnesium matrix during cooling. The onset of dynamic recrystallisation could be observed locally, and was favoured along twins or at the grain boundaries of the original microstructure. Only a slight decrease in flow stress could be observed in the course of the flow curve (Figure 2a). Therefore, it was assumed that it was mainly the hardening processes that occurred. In addition, kink deformation of the LPSO phase occurred (Figure 6a), as shown in Figure 9b at 400 °C. At low forming temperatures, kink band formation accounted for the majority of the plastic deformation [14]. A forming temperature of 350 °C was too low, so that nucleation of DRX was suppressed. With a low DRX content, however, this sample exhibited high levels of flow stress, with a maximum of 170 MPa. For a Mg-5.8Y-2.0Zn-0.3Zr alloy, Lv et al. (2014) [33] show that even at low strain rates, which have a more favourable effect on the recrystallisation behaviour—hardly any areas of dynamic recrystallisation can be detected at 350 °C.

**Figure 10:**
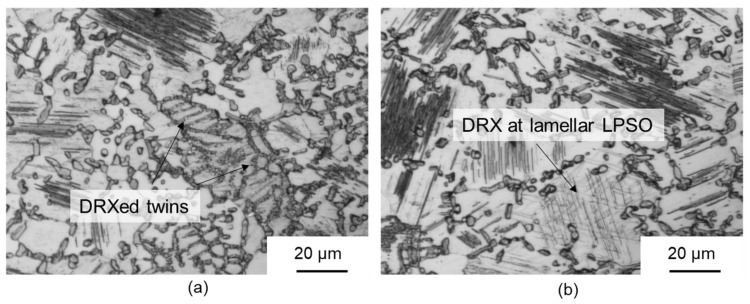
Optical micrographs of deformed samples at 450 °C at an equivalent strain of 0.4 showing different DRX mechanisms: (**a**) DRX at twins and (**b**) DRX at lamellar LPSO phase within matrix grains.

Figure 9b,c shows the microstructure after forming at 400 °C and 450 °C, respectively. Kink deformation of the LPSO phase can also occur here because of the high proportion of lamellar LPSO phases within the grains of the magnesium matrix. Dynamic recrystallisation could be observed in this temperature range at kink boundaries (Figure 9b) and grain boundaries of the original microstructure (Figure 9c). To determine the origin of the dynamic recrystallisation, samples with a low degree of deformation (φ = 0.4) were compressed at 450 °C and analysed by light microscopy (Figure 10). It was found that the nucleation of dynamic recrystallisation at low equivalent strains starts preferentially at twins (Figure 10a) and in grains with lamellae that were plastically deformed by kink deformation (Figure 10b). Thus, several mechanisms of dynamic recrystallisation during forming of the twin-roll cast and heat-treated Mg-6.8Y-2.5Zn-0.4Zr strip could be identified. Nucleation occurred primarily at twin boundaries (twin-induced dynamic recrystallisation, TDRX). Occasionally, DRX occurred at the grain boundaries of the original microstructure, resulting in the characteristic necklace structure during continuous dynamic recrystallisation (CDRX). The network-like LPSO phases, which were present along the original grain boundaries of the magnesium grains, impeded dislocation movement during forming such that an increase in dislocation density and, thus, in stress concentration occurred, and in particular at grain boundaries. Regions not affected by DRX were present within these grains. When the lamellar LPSO phase occurs, CDRX is mainly considered to be a recrystallisation mechanism [14,33,34]. In the present investigations, however, TDRX and DRX were increasingly observed at kink boundaries. The influence of kink deformation and DRX at kink boundaries decreased continuously with increasing forming temperatures, since the proportion of lamellar LPSO phases decreases with increasing temperature. This mechanism disappears completely at 500 °C, since no lamellar LPSO phases occur.

**Figure 11:**
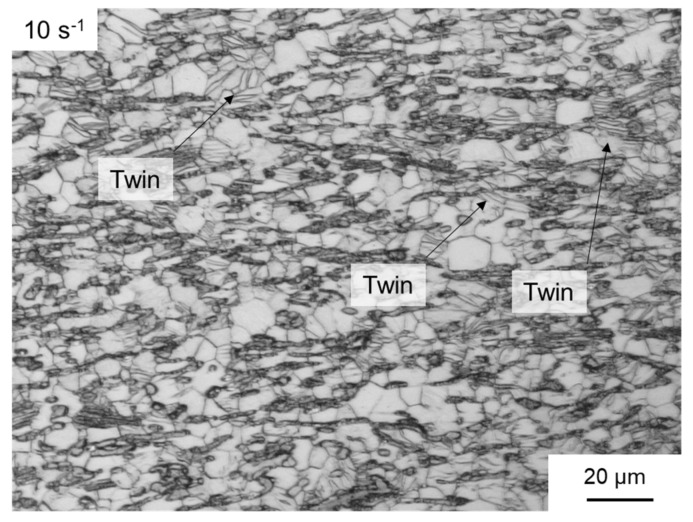
Optical micrograph of sample deformed at 10 s^-1^ and a low equivalent strain of 0.2.

At higher strain rates, twin formation played a more significant role (Figure 9). When forming began (φ = 0.2), high twinning density could already be identified in the microstructure at a strain rate of 10 s^−1^ (Figure 11). In the further course of the forming process, these twins could serve as initiation points for the nucleation of dynamic recrystallisation, as shown in Figure 10a. The high twinning density favoured the course of DRX, as shown previously in Figure 7. Similar results for magnesium alloys with LPSO phases are presented in [14,33].

### 3.6. Processing Maps

**Figure 12:**
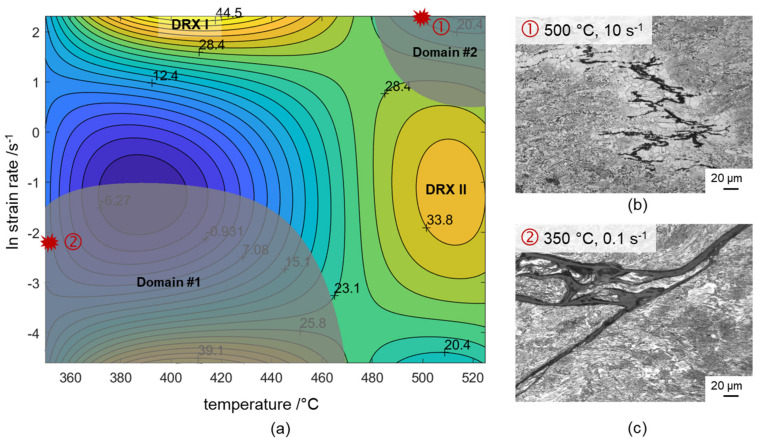
(**a**) Processing map of twin-roll cast and heat-treated Mg–6.8Y-2.5Zn-0.4Zr alloy at an equivalent strain of 0.4 and optical micrographs from areas of instability: (**b**) Domain #2: 500 °C, 10 s^−1^ and (**c**) Domain #1: 350 °C, 0.1 s^−1^ (red circles denote domain, where the micrographs refer to).

A processing map is the superimposition of a power dissipation efficiency map and the instability parameter map. The flow-stress data obtained from the hot compression tests are used to plot the processing maps using different DMM models. These maps can provide the processing window along with an opinion on deformation mechanisms that can be helpful for industrial applications. To avoid accumulation of internal energy, the processing maps are plotted for strains after strain hardening. The corresponding material parameters *m*, *η* and *ξ* are calculated according to [20,21]. Isolines of temperatures and strain rates are plotted on the two-dimensional plane; namely the power dissipation map and the instability map. The two processing maps based on the Prasad instability criterion are obtained by superimposing them. As shown in Figure 12, the shadow zone in the processing map is the instability zone and the coloured zone is the safe machining zone.

According to the processing map, two instability zones could be recognised: the first instability zone occurs in the temperature range of 350 °C to 460 °C and over a strain-rate range of 0.01 s^−1^ to 0.3 s^−1^; the second instability zone occurs in the temperature range of 485 °C to 525 °C and over a strain-rate range of 2.5 s^−1^ to 10 s^−1^. In these zones, the corresponding power dissipation coefficient of materials is comparatively low, and the instability of adiabatic shear bands or local plastic flow tends to occur during deformation. Cracks appeared in the alloy in these instability zones, as can be seen in the micrographs of Figure 12b,c.

Large values of *m* were observed at moderate to high temperatures (460 °C to 525 °C) at strain rates of up to 2.5 s^−1^. Moreover, in this domain the stress value increased steadily to a peak followed by softening towards the steady-state region (see, for example, Figure 2), which is a typical observation made during DRX. This was also proved by optical micrography as shown in Figure 7, which shows recrystallised grains without any cracks, because no flow lines of the plastic deformation are observed. In addition, DRX regions are also visible when the strain rates are increased above 0.3 s^−1^ and the temperatures are decreased to 400 °C or 450 °C. In these ranges, the values of *m* and *η* are high, and stability is predicted. Therefore, such stable ranges offer the best processing parameters.

## 4. Conclusions

In this work, the deformation behaviour of a twin-roll cast (TRC) Mg-6.8Y-2.5Zn-0.4Zr alloy during plane strain compression was characterised by high-temperature testing and the following conclusions can be drawn:

(1) It was assumed that DRX was favoured in the twin-roll cast and heat-treated Mg-6.8Y-2.5Zn-0.4Zr alloy. In contrast to the conventionally cast initial material, there were no lamellae precipitated in the magnesium matrix in the twin-roll cast and heat-treated initial state. Such lamellar LPSO phases can contribute to delay of the DRX process.
(2) The activation energy for the twin-roll cast and heat-treated Mg-6.8Y-2.5Zn-0.4Zr alloy was determined to be 270 kJ/mol, which was significantly higher than that for grain boundaries (92 kJ/mol) or lattice diffusions (135 kJ/mol) in magnesium. The higher value for the activation energy was likely associated with the increased proportion of LPSO phases.
(3) During cooling after heat-treatment temperature to the forming temperature, fine lamellae precipitated within the α-magnesium matrix. Dynamic recrystallisation hardly occurred during forming at temperatures between 350 °C and 400 °C, so that even at an equivalent strain of 1, only a small proportion of recrystallised grains could be observed. One possible reason for this was the precipitation of lamellar LPSO phases, which contribute to the retardation of the DRX process.
(4) Several mechanisms of dynamic recrystallisation during forming of the twin-roll cast and heat-treated Mg-6.8Y-2.5Zn-0.4Zr strip could be identified. Nucleation occurred primarily at twin boundaries (twin-induced dynamic recrystallisation, TDRX). Occasionally, DRX occurred at the grain boundaries of the original microstructure, resulting in the characteristic necklace structure during continuous dynamic recrystallisation (CDRX).
(5) According to the processing map, two instability zones could be recognised: (1) 350 °C to 460 °C and 0.01 s^−1^ to 0.3 s^−1^ and (2) 485 °C to 525 °C and 2.5 s^−1^ to 10 s^−1^. In those areas, the corresponding power dissipation coefficient is relatively low, and also the instability of adiabatic shear bands or nearby plastic flow has a tendency to arise at some stage in deformation.

## Figures and Tables

**Table 1:** Chemical composition of Mg–6.8Y–2.5Zn–0.4Zr (wt.%) alloy as determined by means of optical emission spectrometry (OES).

Y	Zn	Zr	Si	Fe	Cu	Ni	Others	Mg
6.8	2.5	0.4	0.01	0.005	0.001	0.001	0.01	Balance

## Data Availability

Data sharing is not applicable to this article.
